# Determinants of women’s likelihood of vaginal self-sampling for human papillomavirus to screen for cervical cancer in Taiwan: a cross-sectional study

**DOI:** 10.1186/s12905-014-0139-0

**Published:** 2014-11-25

**Authors:** Shu-Ling Chen, Pao-Chun Hsieh, Chia-Hui Chou, Ya-Ling Tzeng

**Affiliations:** Department of Nursing, Hungkuang University, Taichung, Taiwan; Department of Obstetrics and Gynecology, Cheng Ching Hospital, Taichung, Taiwan; School of Nursing, China Medical University, Taichung, Taiwan; Department of Nursing, China Medical University Hospital, Taichung, Taiwan

**Keywords:** Cervical cancer, Pap smear, Human Papillomavirus, Self-sampling

## Abstract

**Background:**

Many Taiwanese women (43.8%) did not participate in regular cervical screening in 2011. An alternative to cervical screening, self-sampling for human papillomavirus (HPV), has been available at no cost under Taiwan’s National Health Insurance since 2010, but the extent and likelihood of HPV self-sampling were unknown.

**Methods:**

A cross-sectional study was performed to explore determinants of women’s likelihood of HPV self-sampling. Data were collected by questionnaire from a convenience sample of 500 women attending hospital gynecologic clinics in central Taiwan from June to October 2012. Data were analyzed by descriptive statistics, chi-square test, and logistic regression.

**Results:**

Of 500 respondents, 297 (59.4%) had heard of HPV; of these 297 women, 69 (23%) had self-sampled for HPV. Among the 297women who had heard of HPV, 234 (78.8%) considered cost a priority for HPV self-sampling. Likelihood of HPV self-sampling was determined by previous Pap testing, high perceived risk of cervical cancer, willingness to self-sample for HPV, high HPV knowledge, and cost as a priority consideration.

**Conclusions:**

Outreach efforts to increase the acceptability of self-sampling for HPV testing rates should target women who have had a Pap test, perceive themselves at high risk for cervical cancer, are willing to self-sample for HPV, have a high level of HPV knowledge, and for whom the cost of self-sampling covered by health insurance is a priority.

**Electronic supplementary material:**

The online version of this article (doi:10.1186/s12905-014-0139-0) contains supplementary material, which is available to authorized users.

## Background

Cervical cancer is the second most common cancer in women worldwide, with over 529,000 new cases and 275,000 deaths in 2010 [1]. Most women with cervical cancer have never been or are not regularly screened [2]. However, cervical screening is crucial for early detection of precancerous lesions. Cervical cancer screening has traditionally been done by physicians using the Pap test, which many women perceive as uncomfortable and embarrassing [3-7]. A potential alternative to the Pap test in Western countries has been vaginal self-sampling for human papillomavirus (HPV) [5,8,9], the causative agent for most cervical cancers [10]. HPV self-sampling is easy to perform, less painful, less embarrassing, and less anxiety provoking than the Pap test [11,12]. Furthermore, HPV self-sampling has been reported to increase cervical cancer screening compliance for women who have never or not regularly been screened for cervical cancer [5,13].

Cervical cancer was the seventh leading cause of cancer mortality among Taiwanese women in 2012 [14]. Taiwan’s National Health Insurance has been reimbursing women ≥30 years old for annual Pap tests since 1995 and women ≥36 years old who had not had a Pap test within ≥6 years for self-collected HPV samples since 2010 [14]. This reimbursement policy led to a gradual but significant increase in annual participation in Pap screening from 35.0% in 1997 to 56.2% in 2011 [14]. Despite this increase, 43.8% of Taiwanese women still have never or not regularly participated in cervical screening in 2011 [14]. Self-collected HPV sampling may improve the cervical cancer screening rate for women who currently do not participate in regular cervical cancer screening in Taiwan, as in western countries [3,13]. However, little research has been done on HPV self-sampling in Taiwan. It is important to identify and target women who are likely to accept self-collected HPV sampling for cervical screening. Thus, the aim of this study was to explore determinants of women’s likelihood of self-sampling for HPV testing.

To explore the likelihood of self-sampling for HPV, we reviewed the literature on factors impacting uptake of HPV vaccination, Pap test, and cervical cancer screening because these factors may also affect uptake of HPV self-sampling. For example, women’s willingness to collect their own HPV samples is crucial [15] because it has been shown to affect their readiness to self-sample for HPV. In addition, two prerequisites for making an informed decision about HPV vaccination are awareness of HPV (having heard of HPV) and knowledge of HPV [16]. The uptake of cervical screening services was influenced by knowledge of cervical cancer [17,18], previous Pap testing [11], educational level [3,11], and household income l [19], but was not associated with marital status [20]. Furthermore, barriers to Pap testing such as the issues of time [20,21], cost [5,18], and clinical site [21] might be barriers to women’s likelihood of self-sampling for HPV.

## Methods

### Sample and setting

This cross-sectional study was conducted between June and October, 2012. A convenience sample was recruited from women seeking obstetrical and gynecological health care but not cervical screening at the gynecologic clinics of a regional hospital in central Taiwan. Women were excluded if they reported a history of cervical cancer because these women received different cervical screening recommendations [22]. After the study was approved by the Cheng Ching Hospital’s Institutional Review Board (approval # HP120013), a clinical nurse approached women in the clinic and told them about the study and their rights. Those who agreed to participate signed informed consent and were given a small gift (NT$ 100 [approximately US$3] cash coupon).

### Data collection

Data were collected by questionnaire from June to October 2012. Women respondents were first asked if they had heard of HPV. If a woman responded “yes”, she was asked three HPV-related questions: her perceived risk of cervical cancer (high/low), her priority considerations for HPV self-sampling (clinical site, time, and cost), and if she had ever self-sampled for HPV (yes/no). If a woman had not heard of HPV, she skipped the first three questions (see “Determinants of the likelihood of self-sampling for HPV” below). All women, regardless of having heard of HPV, were then asked about their willingness to self-sample for HPV (willing, unwilling, undecided) and extent of HPV knowledge. HPV knowledge was measured by 21 true/false items drawn from previous research [23]. Percentages of correct answers for women who had heard of HPV are shown in (Additional file 1). Extent of HPV knowledge was categorized as high, moderate, and low based on the number of correct answers above the mode score, between the median and mode scores, or below the median score, respectively. Scores of ≥15 points were coded as a high level of knowledge, those with 13 to 14 points were coded as moderate knowledge, and those with ≤12 points were coded as low knowledge.

Demographic variables included age range (18–29, 30–39, 40–49, 50–65 years), education (≤ high school, college degree, bachelor’s degree, ≥ graduate degree), annual household income (≤500,000; 510,000-1,000,000; 1,010,000-1,500,000; 1,510,000-2,000,000; ≥2,010,000 NT$), marital status (single [divorced, separated, widowed] vs. married), and residential area (city, town, rural). A related clinical variable was previous Pap testing (yes/no).

### Data analysis

All the data were entered and analyzed using SPSS version 14.0 for Windows (SPSS Inc., Chicago, IL). Data were analyzed by descriptive statistics, the chi-square test, and multivariate logistic regression. All women were asked about their willingness to self-sample for HPV and extent of HPV knowledge because we wanted to determine whether having heard of HPV was associated with willingness to self-sample and HPV knowledge.

## Results

### Sample characteristics

The 500 women who completed our questionnaire were 18–65 years old, with the largest proportion 30–39 years old (*n* = 216, 43.2%). A majority of the women was married (*n* = 372, 74.4%), had at least a college education (*n* = 331, 66.2%), had an annual household income of < NT$1,000,000 (*n* = 397, 79.4%), and lived in a city (*n* = 378, 75.6%). The majority of women had had Pap testing (*n* = 331, 66.2%) and had heard of HPV (*n* = 297, 59.4%). For details, see Table 1.

**Table 1:** **Sample characteristics**

**Characteristics**	***n***	**%**		***n***	**%**
**All women (** ***N*** **= 500)**			**Women who heard of HPV (** ***N*** **= 297)**		
Age, years			Previous Pap testing		
≤29	138	27.6	Yes	206	69.4
30-39	216	43.2	No	91	30.6
40-49	106	21.2	Perceived risk of cervical cancer		
≥50	40	8.0	High	90	30.3
Education			Low	207	69.7
≤High school	169	33.8	Willingness to self-sample for HPV		
College	131	26.2	Willing	196	66.0
Baccalaureate degree	177	35.4	Unwilling/undecided	101	34.0
≥Graduate degree	23	4.6	HPV knowledge^b^		
Annual household income (NT$)			High	76	25.6
≤500,000	194	38.8	Moderate	127	42.8
510,000-1,000,000	203	40.6	Low	94	31.6
1,010,000-1,500,000	67	13.4	Priority consideration for HPV self-sampling		
1,510,000-2,000,000	22	4.4	Cost	234	78.8
≥2,010,000	14	2.8	Clinical site/time	63	21.2
Marital status					
Single (D, S, W)^a^	128	25.6			
Married	372	74.4			
Residential area					
City	378	75.6			
Town	79	15.8			
Rural	43	8.6			
Previous Pap testing					
Yes	331	66.2			
No	169	33.6			
Heard of HPV					
Yes	297	59.4			
No	203	40.6			

^a^D refers to divorced, S refers to separated, W refers to widowed.

^b^HPV knowledge was categorized as high, moderate, and low based on the number of correct answers above the mode score, between median and mode score, or below the median score, respectively. Scores of ≥15 points were coded as “high” knowledge, those with 13 to 14 points were coded as “moderate” knowledge, and those with ≤12 points were coded as low knowledge.

Among the 297 women who had heard of HPV, a majority had had Pap testing (*n* = 206, 69.4%) and perceived that they had a low risk of cervical cancer (*n* = 207, 69.7%). Regarding priority considerations for HPV self-sampling, a majority (*n* = 234, 78.8%) was concerned about cost. Two-thirds of these women were willing to self-sample for HPV (*n* = 196, 66%), and the largest proportion had a moderate level of HPV knowledge (*n* = 127, 42.8%). For details, see Table 1. Most of these women also understood that HPV can cause cervical cancer (97.3%), can cause serious health problems for women (94.9%), cannot be transmitted by kissing (94.3%), and can be sexually transmitted (92.9%). However, few of them knew that using condoms during sexual intercourse is only partially effective in preventing the spread of HPV (8.4%), HPV infection cannot be treated (13.8%), and HPV cannot be transmitted by the exchange of bodily fluids (blood, semen) (14.1%) (Additional file 1).

### Determinants of the likelihood of self-sampling for HPV

If a woman had not heard of HPV, her data were not included in the analysis of determinants of women’s likelihood to self-sample for HPV because not having heard of HPV indicates unawareness of HPV [24]. Associations between demographic/clinical variables and the likelihood of HPV self-sampling among those who had heard of HPV were analyzed using χ^2^ statistics. Significant associations (p < 0.05) were used as the cutoff for selecting determinants in multivariate logistic regression analysis for the likelihood of women’s self-sampling for HPV.

Among the 297 women who had heard of HPV, 69 (23%) had self-sampled for HPV (Table 2). Our analysis showed that, among women who had heard of HPV, ever self-sampling for HPV was significantly associated with five factors: previous Pap testing, perceived risk of cervical cancer, willingness to self-sample, extent of HPV knowledge, and priority considerations for HPV self-sampling. Women who had previous Pap testing and high perceived risk of cervical cancer were more likely to self-sample for HPV than women with no previous Pap testing and low perceived risk of cervical cancer, respectively. Women who were willing to self-sample for HPV were more likely to self-sample for HPV than those who were unwilling and undecided. Women with high HPV knowledge were more likely to self-sample for HPV than those with low and moderate HPV knowledge. Women with a priority consideration of cost were less likely to self-sample for HPV than those with a priority consideration of clinical site/time. In addition, bivariate analyses revealed that, among women who had heard of HPV, ever self-sampling for HPV was not significantly associated with demographic characteristics. For details, see Table 2.

**Table 2:** **Characteristics associated with the likelihood of HPV self-sampling among women who had heard of HPV**

**Characteristic**	**Ever self-sampled for HPV among women who had heard of HPV (** ***N*** **= 297)**		
	**Yes**	**No**	***χ*** ^***2***^
	***n*** **= 69 (23%)**	***n*** **= 228 (77%)**	
	***n*** **(%)**	***n*** **(%)**	
Age range, years			7.54
≤ 29	9 (13.0)	60 (87.0)	
30-39	33 (25.2)	98 (74.8)	
40-49	19 (24.7)	58 (75.3)	
≥ 50	8 (40.0)	12 (60.0)	
Education			1.54
≤ High school	18 (23.4)	59 (76.6)	
College	18 (21.7)	65 (78.3)	
Bachelor’s degree	27 (22.5)	93 (77.5)	
≥ Graduate degree	6 (35.3)	11 (64.7)	
Annual household income (NT$)			7.27
≤ 500,000	16 (16.8)	79 (83.2)	
510,000-1,000,000	32 (24.6)	98 (75.4)	
1,010,000-1,500,000	10 (24.4)	31 (75.6)	
1,510,000-2,000,000	5 (26.3)	14 (73.6)	
≥ 2,010,000	6 (50.0)	6 (50.0)	
Marital status			0.18
Single (D, S, W)^a^	17 (21.5)	62 (78.5)	
Married	52 (23.9)	166 (76.1)	
Residential area			0.14
City	55 (23.6)	178 (76.4)	
Town	10 (22.7)	34 (77.3)	
Rural	4 (20.0)	16 (80.0)	
Previous Pap testing			32.5***
Yes	67 (32.5)	139 (67.5)	
No	2 (2.2)	89 (97.8)	
Perceived risk of cervical cancer			5.85**
High	29 (32.2)	61 (67.8)	
Low	40 (19.3)	167 (80.7)	
Willingness to self-sample			6.03*
Willing	54 (27.6)	142 (72.4)	
Unwilling/undecided	15 (14.9)	86 (85.1)	
HPV knowledge^b^			11.2**
High	28 (36.8)	48 (63.2)	
Moderate	26 (20.5)	101 (79.5)	
Low	15 (16.0)	79 (84.0)	
Priority consideration for HPV self-sampling			
Cost	47 (20.1)	187 (79.9)	6.125*
Clinical site/time	22 (34.9)	41 (65.1)	

^a^D refers to divorced, S refers to separated, W refers to widowed.

^b^HPV knowledge was categorized as high, moderate, and low based on the number of correct answers above the mode score, between median and mode score, or below the median score, respectively. Scores of ≥15 points were coded as a high level of knowledge, those with 13 to 14 points were coded as moderate knowledge, and those with ≤12 points were coded as low knowledge.

*p < 0.05, **p < 0.01, ***p < 0.001.

The results of multivariate logistic regression analyses are presented as factors predicting the likelihood of self-sampling for HPV (Table 3). The results show that the full model, which considered all five independent variables together, was statistically significant (χ^2^ = 72.933, df = 6, *n* = 297, p < 0.001). This result implies that the odds of a woman self-sampling for HPV were related to these five independent variables, i.e., previous Pap testing, perceived risk of cervical cancer, willingness to self-sample, extent of HPV knowledge, and priority considerations for HPV self-sampling. This model also passed tests of goodness of fit and of collinearity. Regarding goodness of fit, the final model passed the Hosmer and Lemeshow test and was not significant (p = 0.945). Each variable also passed tests for collinearity, with all tolerance scores >0.95, which exceeded the suggested criterion of 0.1 [25], and all VIF (variance inflation factor) scores <2, which is well below the cutoff value of 10 [26]. This model correctly classified 78.8% of women who had self-sampled for HPV. The “pseudo” R estimates indicate that the model explained between 21.8% (Cox and Snell R^2^) and 32.9% (Nagelkerke R^2^) of variance in the likelihood of self-sampling for HPV.

**Table 3:** **Logistic regression analysis of determinants for likelihood of HPV self-sampling (** ***N*** **= 297)**

**Determinant**	***β***	**Odds ratio (95% confidence interval)**
Previous Pap testing		
Yes	3.09***	22.00 (5.10, 95.02)
No	1 (ref)^a^	
Perceived risk of cervical cancer		
High	0.81^*^	2.25 (1.17, 4.32)
Low	1 (ref)^a^	
Willingness to self-sample		
Willing	1.13^**^	3.09 (1.41, 6.76)
Unwilling/undecided	1 (ref)^a^	
HPV knowledge		
High (≥70% correct answers)	1.24^**^	3.47 (1.53, 7.88)
Moderate (60%-69% correct answers)	0.22	1.24 (0.58, 2.67)
Low (≤59% correct answers)	1 (ref)^a^	
Priority consideration for HPV self-sampling		
Cost	−1.30^**^	0.27 (0.13, 0.59)
Clinical site/time	1 (ref)^a^	
Constant	−4.39^***^	0.001

^a^ref indicates reference group.

χ^2^:72.933, df = 6, Hosmer & Lemeshow: p = 0.945, −2log likelihood: 249.056, Cox & Snell R^2^: 0.218, Nagelkerke R^2^: 0.329.

*p < 0.05, **p < 0.01, ***p < 0.001.

The odds ratios in Table 3 indicate the factor increases (ratios >1) and decreases (ratios <1) in a woman’s odds of self-sampling for HPV if all other variables are held constant. The strongest determinant of the likelihood of self-sampling for HPV was having had a previous Pap test. Women who had a previous Pap test had 22 times the odds of self-sampling for HPV than those who had not (OR 22.00, 95% CI 5.10-95.02, p < 0.001).

Another strong determinant of women’s likelihood of self-sampling for HPV was priority considerations for HPV self-sampling (Table 3). Women whose priority consideration was cost had significantly lower odds of self-sampling for HPV than those whose priority was clinical site/time considerations (OR 0.27, 95% CI 0.13-0.59, p < 0.01).

Other determinants that significantly contributed to the model were extent of HPV knowledge, willingness to self-sample, and perceived risk of cervical cancer (Table 3). Women with high HPV knowledge had significantly greater odds of self-sampling for HPV than those with low HPV knowledge (OR 3.47, 95% CI 1.53-7.88, p < 0.01). Women who were willing to self-sample for HPV had significantly greater odds of self-sampling for HPV than women who were unwilling/undecided (OR 3.09, 95% CI 1.41-6.76, p < 0.01). Women whose perceived risk of cervical cancer was high had significantly greater odds of self-sampling for HPV than women who perceived a low risk (OR 2.25, 95% CI 1.17-4.32, p < 0.05).

## Discussion

This study contributes to knowledge about the factors related to the likelihood of a clinical sample of Taiwanese women to self-sample for HPV. The majority of our women respondents (59.4%) had heard of HPV, and most of these women (97.3%) understood that HPV causes cervical cancer. However, the remaining 40.6% of our sample had not heard of HPV and did not understand its link to cervical cancer. Although a majority of women had heard of HPV and had moderate-to-high levels of HPV knowledge, they misunderstood important information about the virus, such as condom use only partially protects against transmission of HPV and HPV cannot be transmitted by exchange of bodily fluids (blood, semen). Our results echo those of a study with US college students, most of whom had heard of HPV and had moderate HPV knowledge but did not understand important HPV facts related to cervical cancer [24]. In addition, women who had heard of HPV were more likely to be willing to self-sample for HPV than those who had not heard of HPV. Together, these findings highlight the importance of disseminating information about HPV.

Women’s likelihood of self-sampling for HPV was most strongly predicted by previous Pap testing, consistent with the strongest determinant of intent to undergo future Pap smear screening being prior screening [18]. This result is not surprising, given that most women know and accept the Pap smear as an effective method for cervical screening despite the side effects of discomfort and embarrassment [3,6,27]. In this respect, self-sampling for HPV is a less painful and embarrassing procedure than the Pap test. Thus, women who have undergone previous Pap testing are strongly enough motivated to participate regularly in self-sampling for HPV to prevent cervical cancer or detect it early. We note the discrepancy in our findings between the number of women who had had a Pap test (*n* = 331) and those who had heard of HPV (*n* = 297), suggesting that some women who had a Pap test had not heard of HPV. This may have been due to their doctors not explaining that HPV has been linked to the cytological changes that the Pap test detects or the women not understanding or remembering their doctor’s explanation.

Another strong determinant of women’s likelihood of self-sampling for HPV was cost priority consideration. Cost is a highly negative predictor of self-sampling for HPV. This finding is similar to other studies that vaccine’s cost would impede vaccination [16,28,29].

Three other determinants of women’s likelihood of self-sampling for HPV were extent of HPV knowledge, willingness to self-sample for HPV and perceived risk of cervical cancer. Similar to a previous report that Turkish women’s acceptance of Kato’s device as an alternative to the Pap smear was related to knowledge of HPV and cancer [6]. This result, taken with our finding that even women with moderate-to-high HPV knowledge did not understand some important aspects of HPV knowledge suggest the importance of education programs to increase the likelihood of self-sampling for HPV.

Our findings are consistent with previous findings that willingness to self-sample for HPV was a determinant of future HPV self-sampling [9]. Willingness to self-sample for HPV is a logical predictor for likelihood of HPV self-sampling because without willingness, a health behavior will not occur [30]. Additionally, our clinical sample’s likelihood of HPV self-sampling was predicted by a higher perceived likelihood of getting cervical cancer. This result is consistent with research on acceptability of Kato’s device for cervical cancer screening [6] and low perceived risk of cervical cancer as a major reason for Malaysian women never having a Pap smear [31].

### Limitations

The results of our study are subject to some limitations. First, our results are based on data from a gynecological clinic sample recruited from central Taiwan, and only 69 respondents had ever self-sampled for HPV. Thus, the results may not be generalizable to all women in Taiwan. Second, not having heard of HPV does not mean that a woman has no opinion of her risk of cervical cancer or did not self-sample for HPV. Further research should ask women about their perceived risk of cervical cancer and whether they have ever self-sampled for HPV, regardless of whether they have heard of HPV. Third, determinants of the likelihood of HPV self-sampling were examined only among women who had heard of HPV. More research is needed to find effective methods to disseminate information about HPV to increase the proportion of women aware of HPV and to improve their understanding of the link between HPV and cervical cancer. More accurate and complete knowledge about HPV may increase cervical screening rates among women who do not regularly participate in screening by increasing their willingness to self-sample for HPV as reported for native women in Canada [13].

## Conclusions

Taiwanese women’s likelihood of HPV self-sampling was predicted by having had a previous Pap test, perceiving a high risk of cervical cancer, willingness to self-sample for HPV, a high level of HPV knowledge, and a cost priority consideration. Therefore, outreach efforts to increase the acceptability of self-sampling for HPV should disseminate information about HPV to increase the rate of women who have heard of HPV and target women who not only have had a Pap test, but also perceive themselves at high risk for cervical cancer, are willing to self-sample for HPV, and have a high level of HPV knowledge. Another important concern for HPV self-sampling is that its cost is covered by health insurance. These findings can be used by policy makers to plan appropriate activities and strategies to educate women about HPV self-sampling for cervical screening, as well as to target women who are likely to accept HPV self-sampling, and in turn to increase regular cervical screening participation in Taiwan.

## Additional file

Additional file 1:
**The proportions of correct answers about HPV by women who had heard of HPV.** Correct answers from [23]: Sandfort JR, Pleasant A: Knowledge, attitudes, and informational behaviors of college students in regard to the human papillomavirus. J Am Coll Health 2009, 58(2): 141–149.
