# Supplementary material for: Determinants of women’s likelihood of vaginal self-sampling for human papillomavirus to screen for cervical cancer in Taiwan: a cross-sectional study
Source: BMC Womens Health. 2014 Nov 25;14:139. doi: 10.1186/s12905-014-0139-0 (PMC4253005; doi:10.1186/s12905-014-0139-0)
Supplement: Additional file 1: — The proportions of correct answers about HPV by women who had heard of HPV. Correct answers from [23]: Sandfort JR, Pleasant A: Knowledge, attitudes, and informational behaviors of college students in regard to the human papillomavirus. J Am Coll Health 2009, 58(2): 141–149. [file 12905_2014_139_MOESM1_ESM.docx]

**Appendix. HPV knowledge of women who heard of HPV (*N*=297)**

| Questions about HPV |  | % Correct answers |
| --- | --- | --- |
| HPV can be transmitted by kissing |  | 94.3 |
| HPV can be transmitted by toilet seats |  | 78.4 |
| HPV can cause HIV/AIDS |  | 49.2 |
| HPV can cause herpes |  | 36.4 |
| HPV can cause serious health problems for women^a^ |  | 95.0 |
| HPV can be transmitted by warts on hand/feet touching the genital area |  | 45.6 |
| HPV can be transmitted by sharing underwear or towels |  | 69.7 |
| HPV can cause cervical cancer^a^ |  | 97.3 |
| HPV can be sexually transmitted^a^ |  | 92.9 |
| Most people with HPV have no visible signs or symptoms^a^ |  | 89.6 |
| There is a vaccine to protect against HPV^a^ |  | 84.5 |
| HPV infection can be treated |  | 13.8 |
| HPV can cause infertility |  | 50.2 |
| HPV can be transmitted by genital skin-to-skin contact^a^ |  | 66.7 |
| HPV can be transmitted by oral sex^a^ |  | 51.2 |
| Using condoms during sexual intercourse prevents the spread of HPV |  | 8.42 |
| HPV can cause serious health problems for men^a^ |  | 56.6 |
| HPV can cause genital warts^a^ |  | 67.3 |
| HPV can be transmitted by the exchange of bodily fluids (blood, semen) |  | 14.1 |
| Percentage of sexually active people that acquire HPV |  | 83.2 |
| Most women with HPV will NOT develop cervical cancer^a^ |  | 74.4 |

^a^These statements are true.

(HPV knowledge was measured by 21 true/false items drawn from Sandfort JR, Pleasant A: Knowledge, attitudes, and informational behaviors of college students in regard to the human papillomavirus. J Am Coll Health 2009, **58**(2): 141-49.)
